# Correlation of Surgical Pleth Index with Stress Hormones during Propofol-Remifentanil Anaesthesia

**DOI:** 10.1100/2012/879158

**Published:** 2012-09-02

**Authors:** Xinzhong Chen, Carsten Thee, Matthias Gruenewald, Christoph Ilies, Jan Höcker, Robert Hanss, Markus Steinfath, Berthold Bein

**Affiliations:** ^1^Department of Anaesthesia, Women's Hospital, School of Medicine, Zhejiang University, Hangzhou 310058, China; ^2^Department of Anaesthesiology and Intensive Care Medicine, University Hospital Schleswig-Holstein, Campus Kiel, Schleswig-Holstein, 24105 Kiel, Germany

## Abstract

Eighty patients undergoing elective ear-nose-throat surgery were enrolled in the present study to investigate the relationship between surgical pleth index (SPI) and stress hormones (ACTH, cortisol, epinephrine, norepinephrine) during general anaesthesia which was induced and maintained with propofol and remifentanil using a target-controlled infusion. The study concluded that the SPI had moderate correlation to the stress hormones during general anaesthesia, but no correlation during consciousness. Furthermore, SPI values were able to predict ACTH values with high sensitivity and specificity.

## 1. Introduction

Excessive intraoperative stress evoked by surgical nociceptive stimulation may influence patients' outcome, length of hospital stay, and overall costs of hospital care [1, 2]. To achieve adequate analgesia (antinociception) blunting the intraoperative stress response, most notably haemodynamic instability, it is crucial to use an ideal variable for assessing the stress level or, perhaps more accurately, the balance of nociception-antinociception [3], during general anaesthesia. Unfortunately, such an ideal (continuous, noninvasive, accurate) variable, which may be used for guiding administration of analgesics to attenuate the stress response, is still missing. Traditionally, clinical signs like somatic (movement) or autonomic (tachycardia, hypertension, sweating, and tearing) responses are used to evaluate whether analgesia is adequate, which has been proved to be unreliable demonstrating low specificity [4–7]. 

Recently, the so-called surgical pleth index (SPI), a novel multivariate index using two continuous cardiovascular variables, the normalized heart beat interval (HBI) and the normalized pulse wave amplitude (PPGA), was developed and proposed to quantify the intraoperative stress level or nociception during general anaesthesia [8–10]. Wennervirta et al. [9] found that the SPI performed better than heart rate, blood pressure, or response entropy in detecting nociceptive stimuli during general anaesthesia [11, 12]. Huiku et al. [8] and Gruenewald et al. [10] also showed that the SPI had a negative correlation with remifentanil effect-site concentration (Ce_remi_) and a positive correlation with the intensity of stimulation during total intravenous anaesthesia with propofol and remifentanil. Moreover, one of our previous studies [13] suggested that SPI could be used for guiding remifentanil administration during propofol-remifentanil anaesthesia, which resulted in lower remifentanil consumption, more stable haemodynamics, and fewer unwanted events. Given the results from the above studies, the SPI seems like a relatively ideal variable for measurement of intraoperative stress or nociception.

Stress hormones such as cortisol, adrenocorticotropic hormone (ACTH), epinephrine, and norepinephrine have been validated to evaluate the magnitude of the surgical stress response [14, 15]. The measurement of the above circulating stress hormones, however, requires blood samples and laboratory analysis and is not suitable for bedside monitoring of intraoperative stress [16]. Owing to the absence of a gold standard for evaluating the performance of an indicator of intraoperative stress level, we used the circulating stress hormones as a tool to evaluate the SPI performance in measuring the stress level during general anaesthesia.

In the present prospective, single-blinded, randomized study, we investigated the relationship between SPI values, BIS values, arterial blood pressure (ABP), heart rate (HR) and stress hormones' values (ACTH, cortisol, epinephrine, and norepinephrine) at four event-related time points during propofol-remifentanil anaesthesia in patients undergoing ear-nose-throat (ENT) surgery. We hypothesized that (1) SPI has a different performance at different states of consciousness (loss of consciousness versus consciousness); (2) SPI has a better correlation with stress hormones than ABP, HR, or the BIS.

## 2. Materials and Methods

### 2.1. Patients and Study Design

After obtaining approval of the institutional review board of the University Hospital Schleswig-Holstein, Campus Kiel, and written informed consent, 80 patients ASA physical status I-II, aged 18–70 years, scheduled for elective ear-nose-throat (ENT) surgery expected to last at least 1 h were enrolled in this prospective, single-blinded, randomized trial. Patients with a history of CNS disease (e.g., neurological disorders, head injury, seizure disorders), chronic use of psychoactive medication or abuse of alcohol or illicit drugs, and any clinical significant cardiovascular, renal, hepatic, or endocrinologic disorders were excluded. Patients were randomly allocated to one of two groups: SPI group in which remifentanil titration was guided by SPI values during anaesthesia maintenance or Control group in which remifentanil titration was guided by standard traditional inadequate anaesthesia criteria (Table 1 [17–19]). Some results of this study have been published previously [13].

Patients in both groups were premedicated with 20–30 mg dipotassium clorazepate the evening before and 3.75–7.5 mg midazolam orally 30 minutes before surgery. Surgery always started in the morning to avoid bias caused by the circadian rhythm of circulating stress hormones. On arrival in the operating theatre, an intravenous catheter was inserted into a larger forearm vein, and standard monitoring including noninvasive blood pressure (NIBP), 5-lead electrocardiogram (ECG), and pulse oximetry (SpO_2_) (S/5 Anaesthesia Monitor, GE Healthcare, Helsinki, Finland) was applied. All patients in both groups were monitored with BIS and SPI. After the skin of the forehead had been degreased with alcohol, BIS electrodes (BIS sensor; Aspect Medical Systems, Natick, MA, USA) were positioned as recommended by the manufacturer, and electrode impedance was kept below 7.5 K*Ω* to ensure optimal contact. SPI monitoring shared the same sensor of pulse oximetry which was clamped on the index finger. The calculation of the SPI was done by 10 s intervals and described elsewhere [8]. During the whole period of anaesthesia, NIBP was monitored with an interval of 3 minutes, and SPI, BIS, SpO_2_, and ECG were monitored continuously.

Patients in both groups received target-controlled infusion (TCI) of propofol with a target effect-site concentration (Ce_prop_) of 4 *μ*g·mL^−1^ and TCI of remifentanil with target effect-site concentration (Ce_remi_) of 4 ng·mL^−1^ via target-controlled infusion pumps (TCI; Asena Alaris, Cardinal Health, Basingstoke, UK) and 0.6 mg·kg^−1^ rocuronium before tracheal intubation. For propofol, the pharmacokinetic model of Schnider and colleagues was used [20] and for remifentanil, the pharmacokinetic model of Minto and colleagues [21]. After intubation, the lungs were ventilated to an end-tidal carbon dioxide (ETCO_2_) concentration of 35 (30–40) mmHg. Ce_prop_ was adjusted stepwisely by 0.5 *μ*g·mL^−1^ with 4-minute intervals to maintain BIS level between 40 and 60 (however Ce_prop_ was not reduced below 2 *μ*g·mL^−1^), whereas the Ce_remi_ was not adjusted until start of the surgical procedure.

During anaesthesia maintenance, all patients, irrespective of the group assignment, were kept at BIS level of 40–60 by adjusting the Ce_prop_ stepwisely by 0.5 *μ*g·mL^−1^ with an interval of 4 min.

In the Control group, the Ce_remi_ was adjusted based on traditional signs and symptoms of inadequate anesthesia. Specifically, inadequate anaesthesia was defined as presence of symptoms detailed in Table 1 [17–19] and was treated by increasing the Ce_remi_ by 1 ng·mL^−1^ step wisely until the maximum allowed concentration of 10 ng·mL^−1^. If this was judged insufficient, urapidil 10 mg was given i.v. Hypotension was treated initially by speeding intravenous infusion, then Ce_remi_ was decreased by 1 ng·mL^−1^ stepwisely until the minimum concentration of 4 ng·mL^−1^, and finally, 0.5 ml Akrinor (an intravenous vasopressor, AWD Pharma, Dresden, Germany; 1 mL contain 100 mg cafedrine and 5 mg theodrenaline) was given intravenously. Atropine 0.5 mg was used for bradycardia.

In the SPI group, the Ce_remi_ was adjusted to keep the SPI values between 20 and 50 by increasing or decreasing remifentanil plasma concentration by 1 ng·mL^−1^ step wisely (Ce_remi_ range was limited between 4 and 10 ng·mL^−1^). In case of 20 < SPI < 50, inadequate anesthesia was treated as follows: urapidil 10 mg i.v. for hypertension; Akrinor 0.5 mL i.v. for hypotension; atropine 0.5 mg i.v. for bradycardia. A rescue medication was allowed (propofol bolus of 0.5 mg·kg^−1^) if somatic arousal or a somatic response occurred despite BIS and SPI values within the predefined range.

To facilitate rapid emergence from anesthesia, 15 minutes before the expected end of surgery, PEC_prop_ was reduced in all patients, and a BIS value of over 60 but below 65 was allowed, whereas the PEC_remi_ remained unchanged until the end of surgery. All patients received 0.1 mg·kg^−1^ piritramide for postoperative analgesia. At the end of surgery which was defined as the final surgical suture, both propofol and remifentanil were stopped.

### 2.2. Blood Sampling and Stress Hormones Assay

In both groups, blood samples were taken at four event-related time points (Base: baseline before anaesthesia; Intu: after tracheal intubation; Max: at maximum surgical trauma, defined intraoperatively by the attending surgeon; After-Max: 15 min after the maximum surgical trauma) for measurement of ACTH, cortisol, epinephrine, and norepinephrine. Samples were immediately placed into iced water, cool-centrifuged within 15 min, and stored at −25°C until further analysis. An improved reversed phase high-performance liquid chromatography technique was used for analysis (autosampler: AS 2000, Merck Hitachi, Darmstadt, Germany; pump: L-6200 Intelligent Pump, Merck Hitachi, Darmstadt, Germany; detector: ECD, Merck- LaChrom L 3500 A, Merck Hitachi, Darmstadt, Germany; Analytical Column for separation: RP 18- equilibrated and tested, Bio-Rad Diagnostics, München, Germany; software: D 7000- HPLC- System, Merck Hitachi, Darmstadt, Germany). Reference range of ACTH: 7.2–63.6 pg/mL; cortisol: 6.2–19 *μ*g/dL; epinephrine: <84 pg/mL; norepinephrine: <420 pg/mL.

### 2.3. Statistical Analysis

All data are presented as mean and SD or median and range, as appropriate. GraphPad Prism (Version 5.0, GraphPad Software Inc., San Diego, CA, USA) was used for statistical analysis. For numerical data, statistical analysis was performed with Student's *t*-test (for normally distributed data) and Mann-Whitney *U* test (for nonnormally distributed data) or one-way analysis of variance with Student-Newman-Keuls test (for multiple comparisons); for nominal data statistical analysis was performed by means of a chi-square test. Correlation analysis between variables was performed with Spearman's rank correlation coefficient, and linear regression slopes were analyzed with *F*-test.

Receiver operator characteristics (ROCs) analysis was performed to evaluate and visualize whether SPI was able to indicate a predefined level of a stress hormone blood concentration (we defined the average level of a stress hormone at the time point “Base” as this specific level, because no well-accepted standard of stress hormone exists for denoting a stress state so far, and at the time point “Base” all patients were conscious and could be considered as experiencing kind of stress before induction of anaesthesia). Further, ROC analysis was used to depict threshold values for SPI to indicate the specific hormone level, based on pooled data.

A *P* value of <0.05 was considered as statistically significant.

## 3. Results

All of the 80 patients finished the study and were included into final analysis for demographic data and clinical data such as duration of surgery and duration of anaesthesia. There were no differences between groups with regard to sex, age, height, body weight, duration of surgery, and duration of anaesthesia, duration of surgery, and duration from intubation to start of surgery (Table 2).

Of the 1280 (80 patients, 4 time points, 4 stress hormones) blood samples for stress hormones' assay, 143 samples were excluded from final analysis due to technical issues (such as insufficient amount of blood withdrawn in vial). The remaining 1137 samples were valid for final analysis.

**Figure 1:**
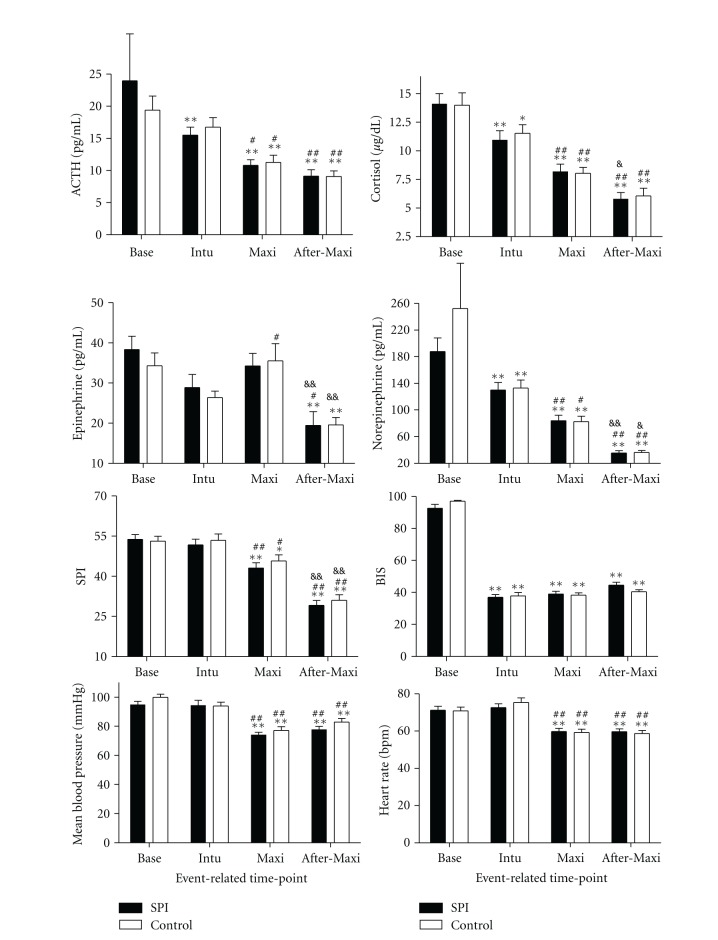
Values are mean ± SD. Base: baseline before anaesthesia; Intu: after tracheal intubation; Max: at maximum operative trauma which was defined intraoperatively by the surgeon; After-Max: 15 min after the maximum operative trauma; Mean: mean blood pressure; HR: heart rate; SPI: the group in which remifentanil administration was guided by SPI; Control: the group in which remifentanil administration was guided by standard traditional inadequate anaesthesia criteria. *P < 0.05, **P < 0.01 compared with respective values at Base in the same group with one-way ANOVA followed by LSD test. ^#^ *P* < 0.05, ^##^ *P* < 0.01 compared with respective values at Intu in the same group with one-way ANOVA followed by LSD test. ^&^ *P* < 0.05, ^&&^ *P* < 0.01 compared with respective values at Maxi in the same group with one-way ANOVA followed by LSD test. No differences were found between the SPI group and the Control group at all four event-related time points (*P* > 0.05).

The plasma concentrations of the stress hormones at the four event-related time points in both groups are presented in Table 3 and Figure 1. In both groups, the plasma levels of all the four stress hormones (ACTH, cortisol, epinephrine, and norepinephrine) decreased from Base to After-Maxi in other words, the plasma levels of the four stress hormones at Base were the highest and at After-Maxi were the lowest among the four event-related time points (Intu, Maxi, and After-Maxi compared with Base, *P* < 0.05). No differences with respect to stress hormone levels at the event-related time points were found between the SPI group and the Control group (*P* > 0.05). 

**Figure 2:**
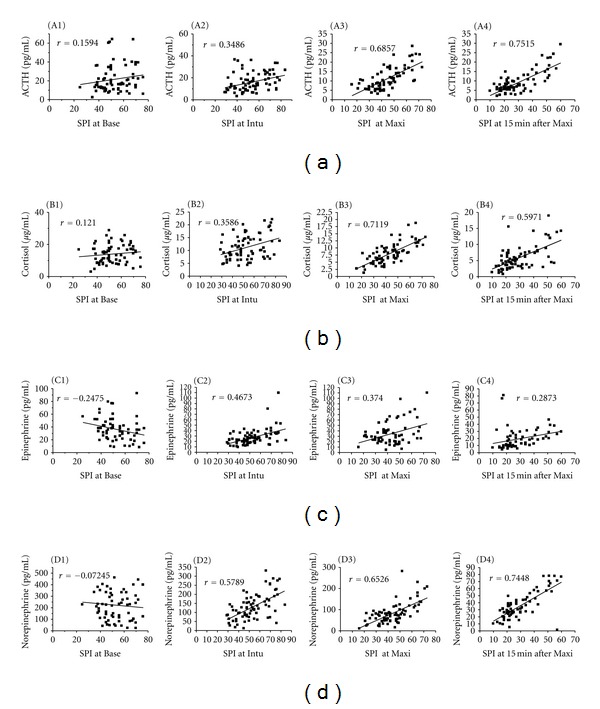
Linear regression analysis and correlation analysis between the SSI values and and stress hormones (ACTH, cortisol, epinephrine and norepinephrine). Bold line is regression line. Slopes of the above lines between time points are significantly different, *P* < 0.01. ACTH: adrenocorticotropic hormone; SPI: surgical pleth index; Base: baseline before anaesthesia; Intu: after tracheal intubation; Max: at maximum operative trauma, defined intraoperatively by the surgeon; After-Max: 15 min after the maximum operative trauma.

Correlations between SPI values and stress hormones (pooled data from both groups) at event-related time points are presented in Figure 2. For all four stress hormones, the absolute values of the correlation coefficient (*r*) at time point Base were the lowest among the four event-related time points (*P* < 0.0001). At the time point Base, values of *r* were less than 0.3, indicating there was no meaningful correlation between SPI values and stress hormones at the time-point Base. Whereas at the other three time points (Intu, Maxi and After-Maxi), absolute values of *r* were between 0.3 and 0.5 (except for the *r* between SPI and epinephrine at the time-point of After-Maxi), indicating that SPI had a moderate or good correlation to stress hormones during general anaesthesia.

Regarding pooled data, SPI could indicate the predefined “specific level” (defined as the average of the data at the time point Base) of ACTH with an area under the curve of 0.85 (*P* < 0.0001), of cortisol with an area under the curve of 0.61 (*P* = 0.00299), of epinephrine with 0.59 (*P* = 0.0198), and norepinephrine with 0.62 (*P* = 0.0143). The optimal threshold value given by ROC analysis for SPI was an SPI ≥46 to predict an ACTH of ≥15 pg/mL with a sensitivity of 81% and a specificity of 73%, the threshold of SPI was ≥44 to predict a cortisol of ≥12 *μ*g/dL with a sensitivity of 66% and a specificity of 53%, the threshold of SPI was ≥46 to predict an epinephrine ≥25 pg/mL with a sensitivity of 59% and a specificity of 53%, and the threshold of SPI was ≥44 to predict a norepinephrine ≥220 pg/mL with a sensitivity of 64% and a specificity of 51%.

## 4. Discussion

In the present study, we investigated the correlation between the surgical pleth index (SPI), a noninvasive variable derived from photoplethysmography which was developed originally for detecting stress or nociception level during general anaesthesia, with stress hormones in the circulating blood in a prospective, single-blinded, randomized study, and demonstrated that (1) SPI had no correlation with stress hormones at the time-point Base, whereas there was moderate-to-good correlation with the stress hormones at the time-points Intu (after tracheal intubation), Max (at maximum surgical trauma defined intraoperatively by the attending surgeon), and After-Max (15 min after the maximum surgical trauma); (2) SPI could predict a specific level of ACTH with high sensitivity and specificity.

It is well known that measuring stress or nociception level during general anaesthesia is very challenging since there are no direct methods to measure it [5, 22]. As a result, some unspecific autonomic reactions, such as blood pressure, heart rate, sweating, or tearing, are used traditionally for clinical evaluation of stress or nociception level during general anaesthesia, which has been proved unreliable with low specificity [5, 6]. Clinical endpoints, such as movement in response to nociceptive stimulation, are commonly used as an indicator of inadequate analgesia [23] but are also unreliable and suppressed by muscle relaxants [24]. Some electroencephalographic (EEG-) derived variables, such as Entropy, especially the difference between state entropy (SE) and response entropy (RE), are proposed to be useful for evaluating the nociceptive component of anaesthesia [17, 25, 26]. Additionally, changes in skin conductivity and photoplethysmographic pulse wave amplitude or pulse wave reflex have been suggested as indicators of stress or nociception [27, 28]. However, the performance of these variables in general is disappointing [29, 30]. Recently, surgical pleth index (SPI) [8], a multivariate index based on the sum of the photoplethysmographic pulse wave amplitude (PPGA) and the normalized heart beat interval (HBI), was developed for measuring the stress or nociception level during general anaesthesia. Several studies showed that the SPI had a negative correlation with remifentanil effect-site concentrations (Ce_remi_) and positive correlation with stimulus intensity during total i.v. anaesthesia with propofol; moreover, the SPI had a better performance in detecting nociceptive stimulation than state entropy (SE), response entropy (RE), heart rate, or PPGA, and the performance in reflecting the stress or nociception level was not influenced by the use of a *β*-blocker (Esmolol) [3, 9, 10, 22, 31]. However, for a newly developed measurement index such as SPI, evaluation of the performance in detecting the stress or nociception level during general anaesthesia should be as comprehensive as possible.

The stress response to surgery is characterized by an increased secretion of pituitary hormones which have secondary effects on hormone secretion from target organs [32]. Hence, ACTH, cortisol, epinephrine and norepinephrine serve as so-called stress hormones, and their blood levels were validated to correlate with intraoperative nociception and used for evaluating the stress or nociception level in surgery [14–16, 33]. It was also proven that excessive secretion of stress hormones was related to worse outcome [1]. In the present study, all four stress hormones were highest at “Base” when all patients were still conscious with some kind of mental stress despite premedication. Moreover, stress hormones were significantly higher at Max which was defined as the time of maximum surgical trauma indicated intraoperatively by the attending surgeon than at 15 min After-Max, indicating that stress hormones reflected well the stress level during general anaesthesia.

Interestingly, we found no correlation between stress hormones and SPI levels at Base, whereas there was a moderate-to-good correlation at the other three event-related time points. As reported, the SPI was developed by Huiku et al. [8] from anaesthetized patients aiming for detecting surgical stress during general anaesthesia. Patients at Base were awake, and may have suffered from preoperative anxiety consequently inducing a stress response, whereas during general anaesthesia patients were unconscious and psychogenic factors unlikely to interfere. Therefore, surgical induced nociception is suggested to be the main factor to elicit a stress response during general anaesthesia. This might be the reason of the discrepancy between correlation of stress hormones and SPI levels at different states of consciousness. Consequently it might be suggested that SPI only works well in anaesthetized patients.

The difference with respect to the magnitude of correlation of the stress hormones with the SPI between the four stress hormones may be explained as follows (1) It is well known that a subject's response to stress, such as surgical stimulation, is characterized by an activation of the hypothalamic-pituitary-adrenal axis, triggering release of ACTH which then stimulates secretion of cortisol from the adrenal cortex. Moreover, stress-induced activation of the hypothalamic-pituitary-adrenal axis HPA axis also initiates sympathetic nervous system production of the catecholamines epinephrine and norepinephrine [34, 35]. The secretion of these stress hormones has a timing sequence and hence at different time point the levels of the stress hormones might be different. In the present study, blood samples for the stress hormones assay were collected between 30 s to 60 s after the event-related time point. This may explain why some stress hormones were already at peak levels whereas other hormones lagged behind. (2) It was shown that responses of stress hormones to surgical stimulation are different [36–38]. Catecholamines such as epinephrine and norepinephrine were reported to be relatively unaffected by the choice of the main anaesthetic drug, and surgical stress caused a similar variation of catecholamines with different inhaled anaesthetics whereas an increase of ACTH and subsequent increase of cortisol are known to correlate well with the severity of surgical stress [37]. Furuya et al. found that plasma concentrations of epinephrine and norepinephrine were within the normal range during surgery whereas plasma concentrations of ACTH and cortisol increased 30 min and 60 min after incision. Further, time to peak differed between hormones. This points to the fact that different stress hormones may have different characteristics with respect to their release after nociceptive stimulation.

Some limitations of the present study should be noted. First, the choice of time points for blood withdrawal may be argued. However, the time points we choose are event related, which referred to nociceptive stimuli level during the course of general anaesthesia. Second, the exact distance between the respective event and blood withdrawal is difficult to define. In our study, we decided to collect blood samples one minute after the event to allow for the release of stress hormones in the circulating blood, while SPI values were noted 15 seconds after the event to allow for the newest data to be displayed on the screen. Third, ENT surgery has lower surgical stimulus relative to other large sugery, which might be the reason that all stress hormones (except epinephrine) were low in the present study. Hence, more styles of surgery are needed to be included in further studies

In conclusion, the SPI showed moderate correlation to the stress hormones (ACTH, cortisol, epinephrine, and norepinephrine) during general anaesthesia, but no correlation during consciousness. Furthermore, SPI values were able to predict ACTH values with high sensitivity and specificity.

## Figures and Tables

**Table 1:** Criteria for inadequate anaesthesia and hypotension or bradycardia.

Inadequate anesthesia	
Hypertension	Mean blood pressure >120% of baseline or >100 mmHg
Tachycardia	Heart rate >90 beats·min^−1^
Somatic arousal	Coughing, chewing, grimacing
Somatic response	Purposeful movement
Hypotension	Mean blood pressure <80% of baseline or <60 mmHg
Bradycardia	Heart rate <80% of baseline or <45 beats·min^−1^

Criteria for inadequate anaesthesia were modified based on previous studies.

**Table 2:** Demographic data.

	SPI (*n* = 40)	Control (*n* = 40)	*P* values
Age (years)	47 ± 17	46 ± 17	0.839
Height (cm)	173 ± 18	171 ± 93	0.657
Weight (Kg)	78 ± 12	75 ± 17	0.438
Gender-M/F (n)	13/27	21/19	0.254
ASA I/II (n)	18/22	19/21	1.000
Duration of anesthesia (min)	152 ± 67	173 ± 84	0.076
Duration of surgery (min)	109 ± 61	132 ± 81	0.108
Intubation to surgery beginning (min)	24 ± 8	25 ± 10	0.534

Values are mean ± SD or absolute numbers. No difference between groups.

SPI: the group in which remifentanil administration was guided by SPI.

Control: the group in which remifentanil administration was guided by standard traditional inadequate anaesthesia criteria.

**Table 3:** Stress hormones levels, mean Bp, heart rate, SPI, and BIS values at the different event-related time points.

Time point	Group	ACTH (pg/mL)	Cortisol (pg/mL)	Epinephrine (pg/mL)	Norepinephrine (pg/mL)	Mean (mmHg)	HR (beat/min)	SPI	BIS
Base	SSI	23 ± 14	14 ± 5	38 ± 18	187 ± 114	94 ± 14	71 ± 13	53 ± 11	92 ± 15
	Control	19 ± 13	13 ± 6	34 ± 18	252 ± 172	99 ± 14	70 ± 12	53 ± 11	97 ± 2
Intu	SSI	15 ± 7**	10 ± 4**	28 ± 19	129 ± 69**	94 ± 20	72 ± 12	51 ± 13	37 ± 10**
	Control	16 ± 9	11 ± 4*	26 ± 9	132 ± 73**	93 ± 16	75 ± 15	53 ± 14	37 ± 13**
Maxi	SSI	10 ± 5^∗∗#^	8 ± 4^∗∗##^	34 ± 17	83 ± 50^∗∗##^	74 ± 10^∗∗##^	59 ± 10^∗∗##^	43 ± 12^∗∗##^	39 ± 11**
	Control	11 ± 65^∗∗#^	8 ± 3^∗∗##^	35 ± 24^#^	82 ± 49^∗∗#^	77 ± 16^∗∗##^	59 ± 10^∗∗##^	45 ± 14^∗#^	38 ± 9**
After-Maxi	SSI	9 ± 6^∗∗##^	5 ± 3^∗∗##&^	19 ± 18^∗∗#&&^	35 ± 19^∗∗##&&^	77 ± 13^∗∗##^	59 ± 9^∗∗##^	29 ± 11^∗∗##&&^	44 ± 11**
	Control	9 ± 5^∗∗##^	6 ± 4^∗∗##^	19 ± 10^∗∗&&^	36 ± 18^∗∗##&^	82 ± 15^∗∗##^	58 ± 10^∗∗##^	31 ± 13^∗∗##&&^	40 ± 7**

Values are mean ± SD. Base = baseline before anaesthesia; Intu = after tracheal intubation; Max = at maximum operative trauma which defined intraoperatively by the surgeon; After-Max = 15 min after the maximum operative trauma; SSI = the group in which remifentanil administration was guided by SSI; Control = the group in which remifentanil administration was guided by standard traditional inadequate anaesthesia criteria.

*P < 0.05, **P < 0.01 compare with respective values at Base in the same group with one-way ANOVA followed by LSD test.

^#^ *P *< 0.05, ^##^ *P *< 0.01 compared with respective values at Intu in the same group with one-way ANOVA followed by LSD test.

^&^ *P *< 0.05, ^&&^ *P* < 0.01 compared with respective values at Maxi in the same group with one-way ANOVA followed by LSD test.

No any differences were found at all four event-related timepoints between the SPI group and the Control group (*P * > 0.05).
